# Recent advances in the understanding of cilia mechanisms and their applications as therapeutic targets

**DOI:** 10.3389/fmolb.2023.1232188

**Published:** 2023-09-14

**Authors:** Masaki Saito, Wataru Otsu, Keiko Miyadera, Yuhei Nishimura

**Affiliations:** ^1^ Department of Molecular Physiology and Pathology, School of Pharma-Sciences, Teikyo University, Tokyo, Japan; ^2^ Department of Biomedical Research Laboratory, Gifu Pharmaceutical University, Gifu, Japan; ^3^ Department of Clinical Sciences and Advanced Medicine, School of Veterinary Medicine, University of Pennsylvania, Philadelphia, PA, United States; ^4^ Department of Integrative Pharmacology, Mie University Graduate School of Medicine, Tsu, Mie, Japan; ^5^ Mie University Research Center for Cilia and Diseases, Tsu, Mie, Japan

**Keywords:** ciliary receptors, ciliogenesis, ciliary transport, ciliopathy, inherited retinal dystrophies, photoreceptor

## Abstract

The primary cilium is a single immotile microtubule-based organelle that protrudes into the extracellular space. Malformations and dysfunctions of the cilia have been associated with various forms of syndromic and non-syndromic diseases, termed ciliopathies. The primary cilium is therefore gaining attention due to its potential as a therapeutic target. In this review, we examine ciliary receptors, ciliogenesis, and ciliary trafficking as possible therapeutic targets. We first discuss the mechanisms of selective distribution, signal transduction, and physiological roles of ciliary receptors. Next, pathways that regulate ciliogenesis, specifically the Aurora A kinase, mammalian target of rapamycin, and ubiquitin-proteasome pathways are examined as therapeutic targets to regulate ciliogenesis. Then, in the photoreceptors, the mechanism of ciliary trafficking which takes place at the transition zone involving the ciliary membrane proteins is reviewed. Finally, some of the current therapeutic advancements highlighting the role of large animal models of photoreceptor ciliopathy are discussed.

## 1 Introduction

The primary cilium is a single immotile microtubule-based organelle protruding into the extracellular space. The ciliary membrane abundantly contains specific membrane receptors and ion channels in its limited surface region, which enables cells to sense the extracellular circumstances. Ciliopathies are a group of inherited diseases involving dysfunction of the cilia arising from variants in genes encoding proteins that localize to the cilia or centrosomes. As most cells possess primary cilia, ciliopathies may exhibit pleiotropic effects resulting in a wide range of anomalies in multiple organ systems. Some of the common features include retinitis pigmentosa, renal cystic disease, polydactyly, situs inversus, mental retardation, hypoplasia of corpus callosum, dandy-walker malformation, posterior encephalocele, and hepatic disease, among others (Baker and Beales, 2009). Nearly 200 ciliopathy genes have been documented [reviewed in Wheway et al. (2019)], some of which encoding proteins that are part of complexes involved in cilia formation, function, and trafficking. As such, phenotypic overlaps may be observed between variants in genes encoding components of protein complexes associated in disease such as Bardet-Biedl syndrome, Meckel syndrome, and nephronophthisis.

The primary cilium is therefore gaining attention due to its potential as a drug discovery target. In this review, we provide an introduction to the cilia, the molecules involved in their functions, and an outlook of the current findings about the primary cilium. Specifically, we will examine ciliary receptors, ciliogenesis, and ciliary trafficking as possible therapeutic targets for ciliopathies. First, the mechanisms of selective distribution, signal transduction, and physiological roles of membrane receptors in primary cilia is reviewed to examine the ciliary receptors as therapeutic targets. The next target is ciliogenesis which is a fundamental mechanism that regulates the structural formation of primary cilia, while its dysregulation is associated with various forms of ciliopathy and cancer. We specifically review the Aurora A kinase, mammalian target of rapamycin, and ubiquitin-proteasome pathways as therapeutic targets to regulate ciliogenesis. Ciliary trafficking is critical in cilia maintenance and involves the ciliary membrane proteins for the transport and turnover of critical proteins at the transition zone. Specifically, we examine the trafficking pathway of photoreceptors to review the machinery and molecules involved in intracellular trafficking as possible therapeutic targets. Finally, we review some of the current therapeutic advancements highlighting the role of large animal models of photoreceptor ciliopathy in developing AAV gene therapy for future translational application in patients.

## 2 Ciliary receptors as the therapeutic targets

The surface area of the primary cilia is only 1/1,000 of the entire cell surface. The cilia function as sensory organelles because they harbor selective G protein-coupled receptors (GPCRs), receptor tyrosine kinases (RTKs), and ion channels on their surfaces. This selective distribution is enabled by a diffusion barrier composed of the transition fibers and Y-links in the transition zone (Reiter et al., 2012; Takao and Verhey, 2016). By sensing extracellular cues, including autacoids, hormones, lights, lipids, mechanical stress, and neurotransmitters, cilia regulate cell fate determination and homeostasis. Dysregulation of ciliary signaling leads to hereditary dysfunction and malformations of various organs, known as ciliopathies, with manifestations of brain malformations, mental disability, skeletal malformations, polycystic kidney disease, and retinal degeneration (Reiter and Leroux, 2017). In this section, by introducing the mechanisms of selective distribution, signal transduction, and physiological roles of membrane receptors in primary cilia, we discuss the prospects of ciliary receptors as therapeutic targets for ciliopathies.

Limited types of RTKs and GPCRs have been identified in primary cilia (Table 1). For example, insulin-like growth factor-1 receptor (IGF-1R), platelet-derived growth factor receptor α (PDGFRα), and transforming growth factor-β receptor (TGF-βR) are ciliary RTKs (Schneider et al., 2005; Clement et al., 2013; Yeh et al., 2013). Dopamine receptors (D_1_R, D_2_R, D_5_R), free-fatty acid receptor 4 (FFAR4), melanin-concentrating hormone receptor 1 (MCHR1), neuropeptide Y receptors 2 and 5 (NPY2, NPY5), rhodopsin, serotonin receptor 6 (5-HT_6_), smoothened (SMO), and somatostatin receptor 3 (SSTR3), in addition to olfactory GPCRs, are ciliary GPCRs (Schou et al., 2015; Wachten and Mick, 2021). In general, GPCRs transduce signals of the Gα_q/11_, Gα_s_, Gα_i_, and Gα_12/13_ signaling pathways. Among these, Gα_q/11_ activates phospholipase C, which further induces the inositol 1,4,5-trisphosphate/Ca^2+^ and diacylglycerol/protein kinase C pathways. Gα_s_ activates adenylyl cyclases (ACs), which produce cyclic AMP (cAMP) and promote the activation of the protein kinase A/Epac pathway. In contrast, Gα_i_ inhibits ACs and suppresses cAMP production. It has been considered that ciliary GPCRs activate Gα protein(s) consistent with the receptors in the extracilia. Intriguingly, recent studies have revealed the possibility that certain GPCRs can mediate cilia-specific signaling pathways that are distinct from extracilia pathways.

**TABLE 1:** List of ciliary membrane receptors.

Receptor type	Functions [distributions]	References
G protein-coupled receptors		
Adrenergic β_2_ receptor (β_2_AR)	Promotes neural excitability in CNS [neuronal cells and pancreatic islets α and β cells]	Yao et al. (2016), Wu et al. (2021)
Dopamine receptors (D_1_R, D_2_R, D_5_R)	[neuronal cells and renal epithelial cells]	Domire et al. (2011), Marley et al. (2013), Gazea et al. (2016)
Free-fatty acid receptor 4 (FFAR4)	Adipogenesis, insulin and glucagon secretion [preadipocytes]	Hilgendorf et al. (2019), Wu et al. (2021)
Galanin receptors (GAL2R, GAL3R)	[neuronal cells]	Loktev and Jackson (2013)
G-protein coupled bile acid receptor 1 (GPBAR1/TGR5)	Promotes bile formation in biliary epithelial cells	Keitel et al. (2010)
Kisspeptin receptor 1 (KISS1R)	Controls neuronal activity of gonadotropin-releasing hormone neuron; Regulates insulin and glucagon secretion (?) in pancreatic islets α and β cell [neuronal cells and pancreatic islets α and β cells]	Koemeter-Cox et al. (2014), Wu et al. (2021)
Melanin-concentrating hormone receptor 1 (MCHR1)	Possibility of depression-resistant, energy homeostasis, and food intake [neuronal cells]	Berbari et al. (2008), Nagata et al. (2013), Kobayashi et al. (2018), Kobayashi et al. (2021b)
Melanocortin 4 receptor (MC4R)	Food intake and body weight control [neuronal cells]	Siljee et al. (2018), Wang et al. (2021)
Neuropeptide Y receptor 2 and 5 (NPY2, NPY5)	Control energy balance [neuronal cells]	Loktev and Jackson (2013)
Orphan receptors (GPR19, GPR63, GPR83, GPR161, GPR175)	GPR88, suppress ciliary D_1_R-mediated cAMP production; GPR161, suppress cilia localization of SMO	Marley et al. (2013), Mukhopadhyay et al. (2013)
Parathyroid hormone receptor (PTHR)	Osteoblast survival and osteogenic gene expression [preosteoblasts, osteocytes, and nucleus pulposus cells]	Zheng et al. (2018), Martín-Guerrero et al. (2020), Tirado-Cabrera et al. (2022)
Prostaglandin E2 receptor 4 (EP4)	Insulin and glucagon secretion [pancreatic islets α and β cells]	Wu et al. (2021)
Purinergic receptors (P2Y_12_R, P2Y_14_R)	[cholangiocytes (P2R_12_R), pancreatic islets α and β cells (P2Y_14_R)]	Masyuk et al. (2008), Wu et al. (2021)
Pyroglutamylated RF-amide peptide receptor (QRFPR/GPR103)	[neuronal cells]	Loktev and Jackson (2013)
Rhodopsin	Light sensing [photoreceptors]	Hsu et al. (2015)
Serotonin receptor 6 (5-HT_6_R)	Possibility of depression-resistant, food intake, learning, and memory [neuronal cells]	Lesiak et al. (2018), Sheu et al. (2022)
Smoothened (Smo)	Cell differentiation and development of various organs [variety of cell types]	Echelard et al. (1993), Briscoe and Thérond (2013), Jeng et al. (2020), Shimada and Kato (2022)
Somatostatin receptor 3 (SSTR3)	Objective learning and memory [neuronal cells]	Berbari et al. (2008), Stanić et al. (2009), Einstein et al. (2010)
Vasopressin receptor, type 2 (V_2_R)	Ciliary cAMP elevation [renal epithelial cells]	Sherpa et al. (2019)
Receptor tyrosine kinases		
Endocrine gland-derived vascular endothelial growth factor (EG-VEGF)	Trophoblast invasion	Wang et al. (2017)
Epidermal growth factor receptor (EGFR)	[cilia of odontoblast and renal epithelial cells]	Ma et al. (2005), Jerman et al. (2014)
Fibroblast growth factor receptor 3 (FGFR3)	Shorten cilia length in chondrocytes	Martin et al. (2018)
Insulin-like growth factor-1 receptor (IGF-1R)	Ciliary resorption, cell growth, and development of various organs	Yeh et al. (2013)
Neurotrophic receptor tyrosine kinase 2 (NTRK2/TrkB)	Cilia localization upon BDGF-stimuli (examined in RPE-1 cells)	Leitch and Zaghloul (2014)
Platelet-derived growth factor receptor α (PDGFRα)	Ciliary resorption	Schneider et al. (2005), Pugacheva et al. (2007)
Transforming growth factor-β receptor (TGF-βR)	Differentiation of cardiomyocytes, migration of mesenchymal stem cells and cancer cells	Clement et al. (2013), Gencer et al. (2017)
Ion channels		
Epithelial sodium channel (ENaC)	[renal epithelial cells]	Raychowdhury et al. (2005)
Polycystin-2/TRPP4	Sensing mechanical and shear stresses; Sensing mechanical stress	Nauli et al. (2003), Raychowdhury et al. (2005)
Transient receptor potential family (TRPC1, TRPV4)	[renal epithelial cilia]	Raychowdhury et al. (2005), Kottgen et al. (2008)

### 2.1 Mechanisms of ciliary localization of receptors

Ciliary GPCRs are trafficked from the cytosol to primary cilia with the aid of intraflagellar transport complex A (IFT-A) and tubby-like protein 3 (TULP3) (Mukhopadhyay et al., 2010). Knockdown of TULP3 decreases ciliary localization of various rhodopsin family (class A) GPCRs (Badgandi et al., 2017). Ciliary localization of GPCRs is also achieved by the presence of ciliary targeting sequences (CTS) in the intracellular third loop and the carboxy-terminus of GPCRs. The Ax[S/A]xQ sequence in the third intracellular loop of 5-HT_6_R, MCHR1, and SSTR3 is essential for the ciliary localization of the receptors (Berbari et al., 2008; Nagata et al., 2013). This consensus sequence is also present in other ciliary GPCRs, including D_1_R, rhodopsin, opsin, and olfactory receptors 52N1, 52N4, and 6V1 (Berbari et al., 2008; Domire et al., 2011). Intriguingly, TGF-βR type 1, an RTK, harbors the A^31^TALQ^35^ sequence (Gencer et al., 2017). However, because the sequence is located in the extracellular region, the mechanism of ciliary targeting remains unclear. An [R/K][I/L]W sequence is located in the third loop of NPY2R and GPR83, whereas the [I/V]KARK sequence is in the intracellular third loop of GPR161 (Loktev and Jackson, 2013; Mukhopadhyay et al., 2013). The VxPx sequence of rhodopsin and the LPG motif of 5-HT_6_R have been identified at the carboxy terminus (Mazelova et al., 2009; Barbeito and Garcia-Gonzalo, 2021). Mechanistically, TULP3 interacts with the Ax[S/A]xQ and [I/V]KARK sequences, together with the KTRKIKP motif of fibrocystin (Badgandi et al., 2017). 5-HT_6_R interacts with RABL2 via the CTS for ciliary localization (Dateyama et al., 2019; Barbeito and Garcia-Gonzalo, 2021). The VxPx sequence comprises a protein complex of small G proteins, Arf4 and Rab11, a Rab11 effector FIP3, and an Arf GTPase-activating protein, ASAP1, which regulates the trafficking of rhodopsin from the trans-Golgi membrane to cilia (Mazelova et al., 2009).

The ciliary GPCRs exit from the cilia upon exposure to their agonists, as demonstrated by D_1_R, GPR161, NPY_2_R, SMO, and SSTR3 (Domire et al., 2011; Nager et al., 2017; Shinde et al., 2020). The BBSome, a stable protein complex composed of nine Bardet-Biedl syndrome (BBS) proteins, BBS1, BBS2, BBS3/ARL6, BBS4, BBS5, BBS7, BBS8, BBS9, and BBS18/BBIP10, is an adaptor between the IFT-B complexes and cargo molecules (Nachury et al., 2007; Liew et al., 2014). Agonist-induced K63-linked ubiquitination (UbK63) and retrograde transport by IFT-B and BBSome contribute to the exit of ciliary GPCRs (Nozaki et al., 2018; Shinde et al., 2020; Zhou et al., 2022). Interestingly, vesicle release from the ciliary tip (exocytosis) is reported as another mechanism of BBSome-dependent exit of ciliary GPCRs (Nager et al., 2017).

The mechanisms underlying the ciliary localization of GPCRs and RTKs have remained largely unresolved. Uncovering these mechanisms would be helpful in understanding the pathogenic mechanisms of ciliopathies.

### 2.2 Signal transduction and physiologic roles of ciliary receptors

The Hedgehog signal is a typical ciliary signal and its functions and regulatory mechanisms have been extensively studied. Hedgehog participates in embryonic organogenesis and tissue homeostasis by controlling the differentiation and proliferation of tissue stem/progenitor cells; it controls the development of various organs at the appropriate time and location, including the brain, heart, lung, esophagus, stomach, breast, liver, gallbladder, pancreas, intestines, and blood vessels (Echelard et al., 1993; Briscoe and Thérond, 2013; Jeng et al., 2020; Shimada and Kato, 2022). Three hedgehog proteins in mammals, Sonic, Indian, and Desert hedgehog, target different cell types via their common receptor, SMO (Ingham and McMahon, 2001). In the absence of Hedgehog, a cilia-localizing 12 transmembrane receptor patched-1 (PTCH1) suppresses the ciliary localization of SMO; hence, SMO is distributed in the extraciliary region (Han et al., 2009; Wong et al., 2009). In this case, the full-length GLI is phosphorylated by protein kinase A (PKA) and subsequently ubiquitinated and proteolyzed to form a GLI repressor (GLI-R) (Pan et al., 2006). The cilia-localizing Gα_s_-coupled receptor, GPR161, represses basal SMO activity through Gα_s_/AC/PKA-dependent GLI-R formation (Mukhopadhyay et al., 2013). In contrast, when Hedgehog binds to PTCH1, it is internalized from the ciliary membrane and degraded in lysosomes, and thus, SMO translocates into the cilium (Rohatgi et al., 2007; Yue et al., 2014). Ciliary SMO activates GLI by dephosphorylating the PKA phosphorylation site via an unidentified phosphatase(s) and phosphorylating other sites through unc-51 like kinase 3 (ULK3) and serine/threonine protein kinase 36 (STK36) (Niewiadomski et al., 2014; Han et al., 2019). Finally, active GLI binds to the consensus sequence GACCACCCA and induces the expression of specific genes that regulate the cell fate of target cells (Hallikas et al., 2006). Defect in ciliogenesis by depleting IFT proteins or membrane-associated cytoskeletal protein 4.1G abolishes cilia-mediated hedgehog signaling, highlighting the physiological importance of hedgehog signaling *in vivo* and *in vitro* (Gazea et al., 2016; Yuan et al., 2016; Eguether et al., 2018; Saito et al., 2022).

Interestingly, extraciliary SMO transduces the Gα_i_ signal. In cilia-deficient preosteoblasts, SMO is widespread in the extraciliary region and inhibits osteoblast differentiation by activating the Gα_i_/RhoA pathway [34]. The ability of SMO to activate Gα_i_ signaling has also been reported in mammary epithelial cells, where SMO promotes cell proliferation via pertussis toxin-sensitive Gα_i2_ signaling (Villanueva et al., 2015). This evidence demonstrates the presence of dual functions of Hedgehog through ciliary and extraciliary SMO, although insufficient evidence has been obtained regarding whether extraciliary SMO activates Gα_i_ signaling in ciliated cells. Similar to SMO, parathyroid hormone receptor (PTHR), a Gα_s_- and Gα_q_-coupled GPCR, translocates into primary cilia upon PTH-related protein treatment and shear stress stimuli; ciliary PTHR activates canonical GLI-dependent cell survival and osteogenic gene expression in osteoblastic and osteoclastic cells (Zheng et al., 2018; Martín-Guerrero et al., 2020; Tirado-Cabrera et al., 2022). Based on these studies, it is presumed that primary cilia have a specialized condition(s) of coupling between GPCRs and their effector proteins, which is distinct from that of the extraciliary region.

RTKs and GPCRs coordinate to control brain development and function. Neural progenitor cells (radial glial cells) of the cortex display primary cilia on the ventricular surface during the G_0_/G_1_ phases of the cell cycle at the embryonic stage. Growth factor stimuli, such as IGF-1, primes ciliary resorption and re-entry of cells into the G_2_/M phases, which leads to the proliferation and differentiation of cells, initiating corticogenesis (Li et al., 2011; Yeh et al., 2013) Mechanistically, Aurora A kinase (AURKA) has been identified as one of the major molecules; a growth factor stimulus elicits HEF1-dependent activation of AURKA, which in turn promotes histone deacetylase 6 (HDAC6) activation and ciliary axonemal destabilization (Pugacheva et al., 2007). Calmodulin, NIMA-related protein kinase 2 (NEK2), and polo-like kinase 1 (PLK1) regulate the AURKA pathway (Lee et al., 2012; Plotnikova et al., 2012; Spalluto et al., 2012). AURKA also assembles actin in cilia and causes decapitation of the ciliary tip membrane to execute ciliary resorption (Phua et al., 2017). In contrast, Tctex-1 is a light chain component of the dynein complex. It plays a dynein-independent role when phosphorylated at Thr94 (phospho-(T94)Tctex-1) and is released from the dynein complex. Activation of ciliary IGF-1R accumulates phospho-(T94)Tctex-1 at the ciliary transition zone, and subsequently, phospho-(T94)Tctex-1 primes Cdc42- and Arp2/3-dependent branched actin organization and clathrin-dependent endocytosis at the ciliary pocket membrane (Li et al., 2011; Yeh et al., 2013; Saito et al., 2017). Currently, there are no reports connecting AURKA- and Tctex-1-mediated mechanisms. In addition to IGF-1R, PDGFRα, a cilia-localizing RTK, also promotes ciliary resorption, indicating a possible contribution of this receptor to brain development (Schneider et al., 2005).

SSTR3, a Gα_i_-coupled receptor, is highly expressed in the CA1 and CA3 regions of the hippocampus and the granular layer of the dentate gyrus in postnatal stages (Stanić et al., 2009). It is considered that CA1 cilia-localizing SSTR3 regulates object learning and memory by activating neuronal cilia-specific AC type 3 (AC3) (Einstein et al., 2010; Wang et al., 2011). MCHR1, a Gα_i_-coupled receptor that is extensively expressed in the brain, controls food intake and energy homeostasis (Shimada et al., 1998; Chen et al., 2002; Marsh et al., 2002). It has been reported that ciliary MCHR1 in the hippocampal CA1 region regulates these functions, as well as depression-resistant behavior (Kobayashi et al., 2018). Shortening of hippocampal cilia through the MCHR1/Gα_i_/Akt pathway is considered to present a possible mechanism of feeding and mood (Kobayashi et al., 2021a). In contrast, 5-HT_6_R in cilia is considered to contribute to depression and eating defects, and learning and memory (Fisas et al., 2006; Svenningsson et al., 2007; King et al., 2008; Sheu et al., 2022). Physiological roles are executed by modulation of the epigenetic state in neuronal cells, stabilization of the neuronal ciliary structure, changes in neuronal dendritic morphology, and physical contact of the 5-HT_6_R-positive cilia with synapses of neuronal axons (the axo-ciliary synapse) (Lesiak et al., 2018; Sheu et al., 2022). Overexpression of 5-HT_6_R can elongate primary cilia, hense careful consideration is required when exogenously tagged 5-HT_6_R is used as a ciliary marker (Lesiak et al., 2018). Moreover, neuropeptide Y receptor 2 (NPY2R) controls the energy balance by inhibiting the ciliary AC through activation of its coupling G_i/o_ protein in hypothalamic neuronal cilia (Loktev and Jackson, 2013). The adrenergic β_2_ receptor (β_2_AR) increases the ciliary cAMP level via G_s_ protein and enhances neuronal excitability in the central nervous system (CNS) (Yao et al., 2016). These functional studies of neuronal ciliary GPCRs suggest that the GPCRs transduce ciliary signaling through trimeric G protein(s). However, it remains unclear how do the neural ciliary GPCRs transduce ciliary signaling differently from extraciliary GPCR signaling. Functions and/or distributions of other neural ciliary GPCRs are listed in Table 1. Further studies are required to understand the physiological roles and signal transduction of neuronal ciliary GPCRs.

Among the four free fatty acid receptors (FFARs), FFAR1/GPR40, FFAR2/GPR43, FFAR3/GPR41, and FFAR4/GPR120, FFAR4/GPR120 are distributed in primary cilia in preadipocytes (Hilgendorf et al., 2019). FFAR4/GPR120 is recognized as a Gα_q_-coupled GPCR, and it is activated by various saturated fatty acids, unsaturated fatty acids, omega-3 fatty acids, and omega-6 fatty acids (Hirasawa et al., 2005; Wu et al., 2013; Kimura et al., 2020). Omega-3 fatty acid docosahexaenoic acid (DHA) activates ciliary FFAR4/GPR120, increases ciliary cAMP production, and promotes the differentiation of preadipocytes to mature adipocytes (adipogenesis), highlighting the role of this receptor in white adipose tissue generation. FFAR4/GPR120 and EP4 increase insulin and glucagon secretion by augmenting ciliary cAMP levels. A possibility that FFAR4/GPR120 transduces Gα_s_ signaling is supported by a recent study which is performed in HEK293 cells (Mao et al., 2023). These studies collectively suggest a possibility that FFAR4/GPR120 couples with Gα_s_ in cilia. β2AR, purinergic receptor P2Y_14_R, and KISS1R are also expressed in pancreatic islets α and β cells (Wu et al., 2021). The functions and/or distributions of these receptors are listed in Table 1.

Polycystin-2 (PC2) is a six-transmembrane protein that can permeate Ca^2+^ into the cilium by sensing flow and shear stress. PC2, a nonselective Ca^2+^ channel, is also known as TRPP1 and a member of the transient receptor potential (TRP) subfamily. Mutation in the *PKD2* gene, which encodes PC2, is causative of 15% of the autosomal dominant polycystic kidney disease (ADPKD) (Heyer et al., 2016). PC2 functions by forming a heterodimer with polycystin-1 (PC1), an eleven-transmembrane protein, through their coiled-coil domains. In renal epithelial cells isolated from PC1-knockout mice, or wild type renal epithelial cells treated with anti-PC1 or -PC2 neutralizing antibodies, PC2 failed to induce ciliary Ca^2+^-influx, showing the importance of heterodimer formation in PC2 function (Nauli et al., 2003). PC2 also forms a heterodimer with TRPV4 and is distributed on the primary cilia of canine kidney epithelial cell line (MDCK; Madin-Darby canine kidney) (Kottgen et al., 2008). Knockdown of TRPV4 failed to induce the mechanical stimulation-dependent elevation of intracellular Ca^2+^. In contrast, knockdown of PC2 abolished normal cyst formation in the zebrafish pronephros, but knockdown of TRPV4 did not disturb the cyst formation (Kottgen et al., 2008). Further study is required to fully elucidate the function of ciliary TRPV4 in kidney cells.

### 2.3 Drugs that target ciliary receptors

While Hedgehog controls the proliferation of normal tissue stem/progenitor cells, aberrant activation of Hedgehog signaling promotes stem cell maintenance, self-renewal, and regeneration of cancer stem cells and drives basal cell carcinoma, bladder cancer, breast cancer, chondrosarcoma, gastric cancer medulloblastoma, pancreatic cancer, and rhabdomyosarcoma (Han et al., 2009; Wong et al., 2009; Jeng et al., 2020; Yang et al., 2020). Vismodegib, sonidegib, and glasdegib are small-molecule SMO antagonists that have been approved by the US Food and Drug Administration (FDA). Vismodegib and sonidegib are used for basal cell carcinoma (Gould et al., 2014; Burness, 2015) and glasdegib is used for acute myeloid leukemia (Norsworthy et al., 2019). In addition, no approved drug targeting ciliary GPCRs has been identified to date. However, it is plausible that some of the drugs act on ciliary GPCRs because agonists/antagonists of certain GPCRs, which are also distributed in primary cilia, have been developed (e.g., β2AR agonists and antagonists, PTHR agonist teriparatide, EP4 antagonist grapiprant). Interestingly, tolvaptan, a selective vasopressin V_2_ receptor (V_2_R) antagonist, is an FDA-approved ADPKD drug (Raina et al., 2022). It slows the decline in estimated glomerular filtration rate of ADPKD (Torres, 2019). V_2_R is distributed in the primary cilia of two renal epithelial cells from the proximal tubule (LLC-KP1 cells) and inner medullary collecting duct (IMCD cells) (Sherpa et al., 2019). Although the ciliary V_2_R can increase the cAMP levels, its physiological roles are unresolved and remain to be investigated in future studies. Numerous antibody drugs recognize RTKs. Among these, teprotumumab, an anti-IGF-1R monoclonal antibody, has been approved for the treatment of active thyroid-eye disease (Douglas et al., 2020), and olaratumab, an anti-PDGFRα monoclonal antibody, has been used for the treatment of advanced soft tissue sarcoma (Shirley, 2017).

It is anticipated that diagnostic, preventive, or therapeutic agents against ciliary receptors for ciliopathies will be developed in the near future. For this, it is important that we understand the physiological roles and regulatory mechanisms of ciliary receptors in more detail.

## 3 Ciliogenesis as the therapeutic target

The structure of primary cilia changes dynamically to regulate the proliferation and differentiation of cells spatiotemporally (Nishimura et al., 2019; Kasahara and Inagaki, 2021). Ciliogenesis is a fundamental mechanism to regulate the structure of primary cilia (Patel and Tsiokas, 2021). Ciliogenesis involves several steps including the transportation of small cytoplasmic vesicles from the Golgi apparatus to the mother centriole which is converted the basal body (Lee et al., 2018; Wu et al., 2018), anchoring the basal body to the plasma membrane via the distal appendage (Pitaval et al., 2017), removal of coiled-coil protein 110 (CP110) from the basal body to initiate axoneme elongation (Spektor et al., 2007; Schmidt et al., 2009; Goetz et al., 2012; Xu et al., 2016), the fusion of the ciliary vesicle with the plasma membrane and transportation of tubulin with various modification such as acetylation and glutamylation to increase axoneme length (Ishikawa and Marshall, 2017; Wloga et al., 2017). Dysregulation of ciliogenesis is associated with various diseases, including cancer and ciliopathy (Shiromizu et al., 2020; Zhao et al., 2023). Modulation of ciliogenesis in non-tumor cells can also be used to regulate the differentiation in response to pathological stimulation, becoming less prone to diseases caused by the stimulation (Nishimura et al., 2021a; Yamakawa et al., 2021). Therefore, targeting of ciliogenesis can be therapeutic to these diseases (Nishimura et al., 2021b; Duong Phu et al., 2021). In this section, we focus on Aurora A kinase (AURKA), mammalian target of rapamycin (mTOR), and ubiquitin-proteasome (UPS) pathways as therapeutic targets to regulate ciliogenesis (Table 2).

**TABLE 2:** Pharmacological agents to stimulate ciliogenesis.

Chemical	Molecular targets	Mechanisms and phenotypes	References
iCRT14	AURKA	iCRT114, an inhibitor of b-catenin, elongated primary cilia of hTERT-RPE cells and reduced the amounts of AURKA and HDAC6 in human clear cell renal cell carcinoma cell lines	Dere et al. (2015)
Bexarotene	AURKA	Bexarotene, an agonist of RXR, elongated primary cilia of VHL-deficient hTERT-RPE1 and reduced the amount of AURKA in RCC cell line. Bexarotene also decreased tumor incidence in a mouse model of RCC xenograft. Other RXR agonists did not elongate the primary cilia, suggesting AURKA as the target of bexarotene	Chowdhury et al. (2018)
Alisertib	AURKA	Long-term treatment of alisertib induced DNA damage response and cellular senescence with elongation of primary cilia in human fibroblasts	Jeffries et al. (2019)
Tubastatin A	HDAC6	Tubastatin A, an inhibitor of HDAC6, increased ciliogenesis and decreased proliferation of human cholangiocarcinoma cell lines. Tubastatin A also inhibited cholangiocarcinoma growth in a rat model. Tubastatin A induced ciliogenesis in a mouse model of ciliopathy	Gradilone et al. (2013), Yang et al. (2014)
NPT-BEZ235	PI3K and mTORC1/2	NPT-BEZ235, a dual inhibitor of PI3K and mTOR, increased ciliogenesis of VHL-deficient hTERT-PRE1 cells and reduced tumor burden in a mouse xenograft model of RCC.	Chowdhury et al. (2021)
Torin 1	mTORC1/2	Torin 1, an inhibitor of mTORC1/2, increased ciliogenesis and a non-proliferation status of hTERT-RPE1 cells	Lim et al. (2020)
Rapamycin	mTORC1	Rapamycin, an inhibitor of mTORC1, elongated primary cilia and inhibited proliferation of DU145, a human prostate cancer cell line. Rapamycin increased ciliogenesis of fibroblasts derived from patients with Lowe syndrome. Rapamycin increased ciliogenesis through upregulation of p27^KIP1^ in proliferating hTERT-RPE1 cells	Takahashi et al. (2018), Madhivanan et al. (2020), Jamal et al. (2020)
Alprostadil	EP2/EP4	Alprostadil, a synthetic analog of PGE1, increased ciliogenesis of NPHP1-defective renal cells and ameliorated tubular dilatation and pronephric cysts in NPH animal models possibly through suppression of RhoA activation and increase of p27Kip1	Garcia et al. (2022)
Genz-667161	Glucosylceramide synthase	Genz-667161, an inhibitor of glucosylceramide synthase, elongated primary cilia and suppressed GM3 in Bbs2-defective kidney epithelial cells. Genz-667161 also attenuated abnormalities in metabolism, olfaction, and retina of Bbs2 KO mice	Husson et al. (2020)

### 3.1 Targeting AURKA signaling

AURKA is a key player in the inhibition of ciliogenesis and is located at the basal body. It is activated by various proteins including neural precursor cells expressed, developmentally downregulated protein 9 (NEDD9) (Pugacheva et al., 2007), centrosomal protein 55 (CEP55) (Zhang et al., 2021), and trichoplein (TCHP) (Inoko et al., 2012). Activated AURKA inhibits ciliogenesis through the phosphorylation of substrates such as histone deacetylase 6 (HDAC6) (Pugacheva et al., 2007; Kim et al., 2014; Sánchez de Diego et al., 2014). Phosphorylated HDAC6 deacetylates α-tubulin and reduces the stability of axoneme microtubules (Pugacheva et al., 2007; Sánchez de Diego et al., 2014). Activation of AURKA and suppression of ciliogenesis are observed in various cancers, including epithelial ovarian cancer (Egeberg et al., 2012), prostate cancer (Qie et al., 2020), pancreatic ductal adenocarcinoma (Li et al., 2003; Kobayashi et al., 2017), and glioblastoma (Duncan et al., 2010; Álvarez-Satta and Matheu, 2018). Inhibition of HDAC6 restores ciliogenesis and suppresses the proliferation of cancer cells, including glioblastoma (Urdiciain et al., 2019), cholangiocarcinoma (Gradilone et al., 2013), and chondrosarcoma (Xiang et al., 2017). These findings suggest that inhibition of AURKA signaling may suppress the proliferation of these tumor cells through stimulation of ciliogenesis (Nishimura et al., 2019; Peixoto et al., 2020).

Inhibition of AURKA signaling can be done in different ways, including inhibition of the kinase activity, decreasing the expression, and targeting proteins that can activate or be activated by AURKA (Bertolin and Tramier, 2020; Nishimura et al., 2021b). Alisertib, also known as MLN8237, is an ATP-competitive inhibitor of AURKA (Sells et al., 2015). iCRT14, a βcatenin-responsive transcription inhibitor, and bexarotene, a retinoid X receptor agonist, can decrease the expression of AURKA (Dere et al., 2015; Chowdhury et al., 2018). These chemicals induce ciliogenesis and may be used as novel therapies for diseases associated with the loss of primary cilia. However, ciliogenesis induced by the inhibition of AURKA may cause premature senescence by preventing the formation of the mitotic spindle in non-tumor cells (Jeffries et al., 2019). Targeting proteins that can bind and activate AURKA in selective cells may be desirable to maximize therapeutic efficacy and minimize side effects. TCHP may be a candidate for this approach because the knockout mice are viable and show resistance to high-fat diet-induced obesity and increased regeneration following skeletal muscle injuries (Yamakawa et al., 2021; Yamakawa et al., 2022). Chemicals can be developed that inhibit the interaction between AURKA and the binding partner or degrade ciliary proteins by proteolysis-targeting chimeras (Janeček et al., 2016; Bagka et al., 2022). Tubastatin-A, an inhibitor of HDAC6, can also be used to treat diseases associated with ciliary defects by inducing ciliogenesis (Gradilone et al., 2013; Yang et al., 2014).

### 3.2 Targeting mTOR signaling

mTOR signaling is also a major player in ciliogenesis. Activation of class I phosphatidylinositol-3 kinase (PI3K) phosphorylates phosphatidylinositol 4,5-bisphosphate (PIP_2_) at the plasma membrane to generate phosphatidylinositol 3,4,5-trisphosphate (PIP_3_) (Sugiyama et al., 2019; Margaria et al., 2020). PIP_3_ binds to the Pleckstrin-homology (PH) domain of the serine/threonine kinase AKT, which recruits AKT to the plasma membrane to be phosphorylated on Thr308 and Ser473 by phosphatidylinositol-dependent protein 1 (PDK1) and mTOR complex 2 (mTORC2), respectively (Manning and Toker, 2017). The phosphorylated AKT phosphorylates various substrates, including tuberous sclerosis complex (TSC) 1/2 and glycogen synthase kinase 3β (GSK3β) (Margaria et al., 2020). The phosphorylated GSK3β suppresses ciliogenesis and the dysregulation of this pathway contributes to various ciliopathy phenotypes (Thoma et al., 2007; Beurel et al., 2015; Conduit and Vanhaesebroeck, 2020). mTOR complex 1 (mTORC1) is also involved in ciliogenesis (Lai and Jiang, 2020). Overexpression of Rheb, an activator of mTORC1, suppressed ciliogenesis induced by glucose deprivation, whereas inhibition of mTORC1 by rapamycin increased ciliogenesis through upregulation of p27KIP1, a cyclin-dependent kinase inhibitor (Takahashi et al., 2018). These studies suggest that inhibition of mTOR signaling may also be therapeutic by stimulating ciliogenesis.

NPT-BEZ235, a dual inhibitor of PI3K and mTOR, increased ciliogenesis of von Hippel Lindau (VHL)-deficient human telomerase reverse transcriptase (hTERT)-immortalized retinal pigment epithelial (hTERT-RPE1) cells (Chowdhury et al., 2021). NPT-BEZ235 also reduced tumor burden in a mouse xenograft model of VHL-null renal cell carcinoma (RCC) (Chowdhury et al., 2021). Torin 1, an inhibitor of mTORC1/2, increased ciliogenesis and a non-proliferation status of hTERT-RPE1 cells (Lim et al., 2020). Rapamycin, an inhibitor of mTORC1, increased the ciliogenesis of fibroblasts derived from patients with Lowe syndrome, a ciliopathy caused by mutation of OCRL1 (Madhivanan et al., 2020). Rapamycin also elongated primary cilia and inhibited proliferation of DU145, a human prostate cancer cell line (Jamal et al., 2020). The combination of rapamycin and rosuvastatin alleviated the abnormal phenotypes of the patient-derived fibroblasts (Madhivanan et al., 2020). Rapamycin increased ciliogenesis through the upregulation of p27KIP1, a cyclin-dependent kinase inhibitor, in proliferating hTERT-RPE1 cells (Takahashi et al., 2018). p27KIP1 stimulates ciliogenesis through the regulation of the docking of preciliary vesicles to the distal appendage of the basal body (Yukimoto et al., 2020). p27KIP1 is also involved in the ciliogenesis stimulated by alprostadil, a synthetic analog of prostaglandin E1 (Garcia et al., 2022). mTORC2 increases the synthesis of glycosylceramide (Guri et al., 2017). Genz-667161, an inhibitor of glycosylceramide synthase, alleviated multi-organ pathology in Bardet-Biedl syndrome mice through increasing ciliogenesis (Husson et al., 2020).

### 3.3 Targeting UPS signaling

The ubiquitin-proteasome pathway also regulates ciliogenesis through the control of the proteolysis of ciliary proteins (Hossain and Tsang, 2019; Shiromizu et al., 2020; Habeck and Schweiggert, 2022). Protein ubiquitination occurs in three steps, including binding ubiquitin to ubiquitin-activating enzymes (E1), transfer of the ubiquitin to ubiquitin-conjugating enzymes (E2), and ligation of the ubiquitin to lysine residues on the target protein. The selectivity of target protein ubiquitination is conferred by the combination of E2 and E3 enzymes. Deubiquitinases (DUB) remove the ubiquitin moieties from ubiquitinated proteins (Leznicki and Kulathu, 2017). For example, TCHP is ubiquitinated and deubiquitinated by CRL3-KCTD17 and ubiquitin-specific peptidase 8 (USP8), respectively (Kasahara et al., 2014; Kasahara et al., 2018). Knockdown of KCTD17 shortened primary cilia of hTERT-RPE1 cells through inhibition of proteolysis of TCHP, whereas knockdown of USP8 increased the ciliogenesis through stimulation of proteolysis of TCHP (Kasahara et al., 2014; Kasahara et al., 2018). IQ motif containing B1 (IQCB1), also known as NPHP5, is ubiquitinated by membrane-associated ring-CH-type finger 7 (MARCHF7) and tripartite motif containing 32 (TRIM32) and deubiquitinated by ubiquitin-specific peptidase 9X (USP9X) (Das et al., 2017a). CP110 is ubiquitinated and deubiquitinated by SCF-CyclinF and ubiquitin-specific peptidase 33 (USP33), respectively (D'Angiolella et al., 2010; Li et al., 2013). These findings suggest that targeting E3 ubiquitin ligases and DUBs that regulate ciliary protein may be used to treat cilia-related diseases (Shiromizu et al., 2020).

Scientific advances in the mechanisms for cell type-specific ciliogenesis increase the possibility to develop therapeutic drugs for diseases caused by the impairment of ciliogenesis.

## 4 Intracellular trafficking pathway to primary cilia

The cilium contains a distinct composition of lipids, transmembrane, and membrane-associated proteins which are regulated by the transition zone at the base of the cilium. Since the primary cilia do not have a translational system, ciliary membrane proteins are supplied from the cytoplasm. Mutations in approximately 200 genes have been identified to be linked to ciliopathies (Reiter and Leroux, 2017), and a variety of ciliopathies are caused by the mistargeting of ciliary membrane proteins. Thus, the transport and turnover of receptors located on the cilia have been investigated as clinical interests. While the mechanisms of ciliary transport have been reviewed extensively by others (Malicki and Avidor-Reiss, 2014; Mukhopadhyay et al., 2017; Blacque et al., 2018; Witzgall, 2018; Long and Huang, 2019; Sánchez-Bellver et al., 2021), herein we describe the recent findings from the ciliary transport studies and provide insights into the therapeutic approaches.

The secretory pathway via vesicles that carry ciliary membrane proteins has been well-studied in rhodopsin transport (Wang and Deretic, 2014). Rhodopsin is a GPCR responsible for visual signals in photoreceptors. The photoreceptor is an excellent model for studying ciliary trafficking as the rods have exaggerated cilia, termed the outer segment (OS). OS-specific proteins such as the rhodopsin reaches the destined localization through the connecting cilium, which is equivalent to the transition zone of the primary cilium. The C-terminus of rhodopsin retains a CTS motif sequence (VxPx) that is sufficient for its targeting to OS. The general idea is that membrane-integrated proteins are newly synthesized at the endoplasmic reticulum (ER) and transported through the Golgi apparatus and the *trans-*Golgi network (TGN). The trafficking of newly synthesized rhodopsin from the TGN to the cilium is facilitated by nucleating the formation of a series of protein complexes called rhodopsin transport carriers (RTCs) including Arf4. Arf4-GTP binds to the VxPx motif of rhodopsin (Deretic et al., 2005) and coordinates the transport of rhodopsin in transport vesicles together with ankyrin repeat and PH domain 1 (ASAP1), Rab11-GTP, and Rab11 family interacting protein3 (FIP3) (Wang et al., 2012; Wang and Deretic, 2014). After the release of Arf4 from the complex, the C-terminal cytoplasmic tail of rhodopsin associates with the dynein light chain Tctex-1, which allows RTCs to traffic from the Golgi apparatus to the base of the cilium (Tai et al., 1999). These post-Golgi rhodopsins transit through Rab11-positive recycling endosomes, and preferentially enter the OS in the dark (Hsu et al., 2015). The C-terminal OS targeting signal of rhodopsin partially overlaps with the binding site for visual arrestin, thus the interaction between photoexcited rhodopsin and visual arrestin could contribute to retaining OS entry under light stimulation. Further investigation is required to establish this proposed model on the molecular mechanism underlying the rhodopsin OS entry regulated by light.

On the other hand, some other integral membrane proteins utilize an unconventional intracellular trafficking pathway that bypasses the Golgi apparatus on their way to the primary cilium (Witzgall, 2018). One example is peripherin-2/rds (PRPH2), a photoreceptor-specific tetraspanin protein concentrated in the OS that is essential for its development and structure (Travis et al., 1989). *N*-glycans attached with PRPH2 isolated from rod OS is still sensitive to endoglycosidase H (Travis et al., 1991; Tian et al., 2014). Pharmacological inhibition of the transport via the Golgi apparatus had no effect on the distribution of PRPH2 at the primary cilium (Tian et al., 2014). The C-terminus of PRPH2 contains the lysine residues for ubiquitination and binding to Hrs, a component of the endosomal sorting complex required for transport (ESCRT) complex which functions to be targeted to the late endosomal, and then to cilia (Otsu et al., 2019). Of note, the murine rod OS exhibits a complementary periodic pattern composed of PRPH2-rich discs and rhodopsin-rich discs (Hsu et al., 2015), suggesting that these proteins could be transported to the OS independently. Another example is polycystin-2 (PKD2), whose mutations are linked to autosomal dominant polycystic kidney diseases characterized by the lifelong formation of fluid-filled cysts originating from parts of the nephron and collecting ducts, leading to renal failure. *PKD2* mutations account for 15% of the autosomal dominant polycystic kidney diseases whereas 85% of the disease is caused by *PKD1* mutations (Mochizuki et al., 1996). The structure of PKD2 is similar to that of the TRP (transient receptor potential) channel family and is distributed to the ER and primary cilia. Interestingly, polycystin-2 bypasses the TGN on its way to the cilium while retaining the sensitivity to endoglycosidase H (Cai et al., 1999; Hoffmeister et al., 2011). In addition, the endosomal network regulates the intracellular transport of polycystin-2 via the interaction of a retromer component with the N-terminus of polycystin-2 (Feng et al., 2017), suggesting that the endocytic pathway has some roles in this unconventional transport to the primary cilia. These unconventional pathways can be a therapeutic target to modulate the ciliary expression of specific cargos.

### 4.1 The protein machinery involved in the ciliary destinations

As described in the previous section, the CTS has been found in ciliary proteins including rhodopsin (Deretic et al., 1998), polycystin-2 (Geng et al., 2006), the cyclic nucleotide-gated channel CNGB1 (Jenkins et al., 2006) as well as soluble protein ADP-ribosylation factor-like 13B (ARL13B) (Nozaki et al., 2017) and inositol polyphosphate-5-phosphatase (INPP5E) (Humbert et al., 2012). The interaction of CTS with binding partners has been investigated and attracted attention as a potential therapeutic target. Rab GTPase and Rab-like membrane trafficking proteins play a crucial role in endomembrane organization and have been linked to cilia-related processes (Blacque et al., 2018). Ciliary membrane trafficking of RTCs is mediated by the Arf4-Rab8-Rab11 cascade (Mazelova et al., 2009; Wang et al., 2012). Recently, Mahajan et al. (2023) reported that ARL13B, an ARF/Arl-family GTPase, has the ciliary targeting sequence at the C-terminal stretch of 17 amino acids containing the RVEP motif, which binds to Rab8-GDP and TNPO1 simultaneously. It has been reported that Rab29 interacts with Rab8, Rab11 and IFT20, and is required for TCR recycling in Jurkat T cells (Onnis et al., 2015). Rab23 is involved in the lateral transport of the D1 dopaminergic receptor from the plasma membrane into the ciliary membrane together with IFT-B and KIF17 (Leaf and Von Zastrow, 2015). The mutations in Rab28 have been implicated in the degenerative eye disease known as autosomal recessive cone-rod dystrophy characterized by an early onset progressive photoreceptor loss (Roosing et al., 2013). Rab28 is a conserved cilium-associated component that has been linked to ciliary transport machinery and is associated with the periciliary membrane, behaving with the IFT (Jensen et al., 2016). Rab28 is also proposed to serve cell non-autonomous functions as a regulator of releasing ciliary ectosomes carrying glial cell morphogenic factors in nematodes (Wang et al., 2014; Jensen et al., 2016; Blacque et al., 2018). Turn et al. (2022) identified new roles of ARF GAPs, ELMOD1, and ELMOD3, in protein trafficking from the Golgi to cilia.

IFT complexes are important for the anterograde and retrograde movement of proteins in primary cilia. IFT-A is critical for retrograde transport driven by a dynein-2 motor, while IFT-B complexes are required for anterograde transport driven by a kinesin-2 motor. IFT interacts with DGKδ (Ding et al., 2017). In Bardet-Biedl syndrome, it has been shown that BBSome plays a crucial role in ciliary trafficking (Jin et al., 2010; Wiens et al., 2010). It mediates the ciliary entry of SSTR3, while it is required forthe signal-dependent exit of the dopamine receptor (Drd1), SSTR3, Gpr161, and PTCH1 from cilia. TULP3, a ciliary protein (Mukhopadhyay et al., 2010), is essential for the transport of cargos including polycystin-2 (Badgandi et al., 2017). Chemicals with the activities which modify these types of machinery can be therapeutic targets.

### 4.2 The regulation of phosphoinositol composition in ciliary membrane

The lipid composition of primary cilia is substantially different from the other part of the cell body. The lipid compositions in the ciliary membrane were regulated by the recruitment of ciliary phosphoinositide phosphatase, INPP5E. INPP5E maintains the phosphoinositide PI(4,5)P2 at a lower level at the ciliary membrane in mammalian cells. INPP5E localizes to the photoreceptor IS and CC, but not to the OS, and retina-specific KO for INPP5E exhibits a rapid rod-cone generation resembling Leber congenital amaurosis (LCA) (Sharif et al., 2021). The interaction between INPP5E and ARL13B is essential for their ciliary membrane retention but is dispensable for its entry into cilia (Qiu et al., 2021). Moreover, ARL13B is required for retinogenesis and the morphogenesis of photoreceptor OS discs (Dilan et al., 2019). Recently, Palicharla et al. (2023) revealed that the tubby domain of TULP3 directly interacts with the amphipathic helix structure of ARL13B, which mediates the trafficking of ARL13B itself as well as lipidated cargos. On the other hand, this important role of ARL13B in the delivery of ciliary cargos can be uncoupled from either the ciliary localization of ARL13B and its regulation of Shh signal transduction (Gigante et al., 2020), suggesting that ARL13B may be involved in the ciliary transport outside of the cilium. Ciliary membrane-associated proteins contain the covalent attachment of a lipid-like farnesyl or a geranylgeranyl group, in which post-translational modifications occur at the ER surface. The transport of lipidated cargo proteins to the cilium is assisted by lipid-binding proteins. These proteins function as trafficking chaperones in which a hydrophobic pocket binding to the lipid fraction enables the lipidated protein to be delivered into the cilium. Two proteins, PDE6D, and UNC119B, have been identified. Once the lipidated proteins undergo post-translational modification at the ER, they form a soluble complex with the trafficking chaperones and transport toward the cilium. ARL3-GTP which is activated by ARL13B is recruited to the complex, and the lipidated cargo is released and associated with the ciliary membrane (Fansa et al., 2016; Hanke-Gogokhia et al., 2016). These interactions can be a therapeutic target to facilitate or block the ciliary transport of specific cargos.

### 4.3 Protein transport into cilia through the diffusion barrier

Lateral diffusion is one of the ways for ciliary targeting after the transport vesicle with ciliary membrane protein fused with the somatic plasma membrane and moves up to the primary cilia in case of smoothened (Milenkovic et al., 2009). Early transmission electron microscopy (TEM) studies of vertebrate photoreceptors have revealed a specialized structure between the inner segment and ciliary OS named “connecting cilium” by Eduardo De Robertis (1956) (de Robertis, 1956). The connections between microtubule doublets and the ciliary membrane exhibit Y-shaped structures (Tokuyasu and Yamada, 1959; Gilula and Satir, 1972). The mouse model clarified that retinitis pigmentosa GTPase regulator (RPGR) is localized to the photoreceptor transition zone, and mandatory for the distribution of rhodopsin in the OS. The diffusion barrier is the membrane portion of the ciliary transition zone, which plays a crucial role in maintaining the specific compositions of proteins and lipids in the ciliary membrane from the plasma membrane (Hu and Nelson, 2011). The final step of lateral transition remains to be elucidated. Septin 2 (SEPT2), a member of the septin family of guanosine triphosphatases, is localized at the base of the ciliary membrane and plays a crucial role in retaining receptors in the ciliary membrane (Hu et al., 2010). Chemicals for the cytoskeleton would remove these barriers and can improve ciliary transport.

In recent years, gene mutations involved in ciliopathies have been found, and many ciliary motif sequences and associated molecular machinery have been identified. Even though recent findings provide valuable insight into the molecular and cell biological mechanism underlying the trafficking of ciliary membrane proteins, the mechanisms are still not fully understood. Since the event of trafficking occurs intracellularly, only small molecules that penetrate the cell membrane are potentially available as therapeutic candidates. Moreover, in case of loss of function gene mutation, modifying the transport itself does not guarantee the functionality of the mutants. Gene therapy, especially the replacement of a faulty gene seems to be a promising approach to the treatment of ciliopathies. We will discuss the strategies of gene therapies for ciliopathies and their recent progress in the next section.

## 5 Ciliopathy models and recent therapeutic advances for inherited retinal diseases

Animal models, both naturally occurring and transgenic, have informed on the molecular basis, pathogenesis, and hence into potential therapeutic targets of a multitude of hereditary diseases. Mammalian models, in particular larger models such as canine, porcine, and primates that better mimic human organs and conditions, have been instrumental in bringing new therapies to patients. Inherited retinal diseases (IRDs) which typically manifest as progressive blinding conditions are prime examples of successful translational development in recent years. While IRDs are broadly recognized to affect vision through the dysfunction of certain cell types, primarily the photoreceptors and the retinal pigment epithelia, certain subcategories of IRDs have been recognized to be ciliopathies. Herein we will review the preclinical and clinical developments of photoreceptor ciliopathy IRDs.

### 5.1 Photoreceptor ciliopathy IRDs and associated genes

The landscape of ciliopathy IRD genes has been reviewed previously (Estrada-Cuzcano et al., 2012; Chen et al., 2021), and our knowledge regarding the pool of known genes and their genotype-phenotype association continues to expand. To date, 281 genes have been associated with IRDs (RetNet, accessed 8/18/23, https://web.sph.uth.edu/RetNet/) of which a considerable fraction is understood to be photoreceptor ciliopathy genes. Clinically, they manifest as broad and often overlapping category of non-syndromic IRDs encompassing retinitis pigmentosa (RP), cone-rod dystrophy (CRD), cone degeneration (CD), and LCA as well as syndromic IRDs such as Bardet-Biedl syndrome (BBS). In most non-syndromic IRDs, primary deficiency associated with the cilia leading to photoreceptor degeneration and hence vision loss in the patient was not suggested until the gene was identified and the localization or function of its protein product was suggested in photoreceptor cilia. While the IRD phenotype in syndromic ciliopathy is explained by the expression in multiple cell types of the disease-associated gene product, the non-syndromic ciliopathy IRDs suggest a cell-type specific role of the disease-associated gene product or that of its modifier, if present.

### 5.2 Canine models of photoreceptor ciliopathy IRDs

While simple organisms such as *C. elegans* have contributed to the understanding of the biology and pathophysiology of cilia, therapeutic advancement which targets ciliopathy has been further accelerated through the development of mammalian models. Generation and/or characterization of murine models of IRDs most of which are transgenic have allowed perhaps the most comprehensive understanding of genetic association with IRD phenotypes. Meanwhile, murine models of IRDs often disadvantaged themselves by poorly recapitulating the human phenotype. There was also variable therapeutic relevance due to anatomical differences such as the globe size and the distribution of photoreceptor cell types across the retina. Phenotypic consistency of the murine models is an advantage allowing for the reliable evaluation of therapeutic outcomes. Still, there exceptions of some forms of IRD in which phenotypic variability has been documented to be affected by the genomic background of the murine strain carrying the primary causal genetic variant (Mollema et al., 2011).

Large animal models of IRD, the most notable of which may be naturally occurring canine models which have contributed to the understanding of the molecular basis (Miyadera et al., 2012), pathogenesis, and therapeutic development. To date, at least 53 different forms of canine IRDs have been identified at the molecular level, of which 18 are found to be ciliopathy IRDs (Table 3).

**TABLE 3:** Ciliopathy IRDs in large animals and their associated genes/human diseases.

Gene symbol	Animal disease	Species	Human disease	References
*BBS2*	Syndromic PRA	Dog	BBS	Hitti-Malin et al. (2021)
*BBS4*	PRA	Dog	RP	Chew et al. (2017)
*BBS7*	NHP	NHP	BBS	Peterson et al. (2019)
*C2orf71*	PRA-rcd4	Dog	RP	Downs et al. (2013)
*CCDC66*	PRA	Dog	RP	Dekomien et al. (2010)
*CCDC66*	Early-onset PRA (EOPRA)	Dog	RP	Murgiano et al. (2020)
*CEP290*	PRA	Cat	RP	Menotti-Raymond et al. (2007)
*FAM161A*	PRA, PRA3	Dog	RP	Downs and Mellersh (2014)
*IFT122*	PRA	Dog	RP	Kaukonen et al. (2021)
*MAP9*	PRA-cord1 modifier	Dog	CRD, LCA	Forman et al. (2016)
*NPHP4*	PRA-crd	Dog	CRD	Wiik et al. (2008)
*NPHP5*	PRA-crd2	Dog	LCA	Goldstein et al. (2013)
*NPHP5*	EOPRA	Cat	Senior-Loken syndrome (LCA)	Oh et al. (2017)
*RPGR*	PRA, XLPRA1	Dog	XLRP	Zhang et al. (2002)
*RPGR*	PRA, XLPRA2	Dog	XLRP	Zhang et al. (2002)
*RPGR*	PRA, XLPRA	Dog	XLRP	Kropatsch et al. (2016)
*RPGRIP1*	PRA-cord1, PRA-crd4	Dog	CRD, LCA	Mellersh et al. (2006)
*TTC8*	PRA, GR-PRA2	Dog	BBS	Downs et al. (2014)

BBS, Bardet-Biedl syndrome; RP, retinitis pigmentosa; CRD, cone-rod dystrophy; LCA, leber congenital amaurosis; PRA, progressive retinal atrophy; XLRP, X-linked retinitis pigmentosa.

Therapeutically, canine models have paved the way for new AAV-based gene therapies to be translated into clinical applications of ciliopathy IRDs. Beltran et al. (2012) evaluated gene augmentation therapy in canine models of X-linked RP (XLRP) caused by variants in *RPGR* which encodes a photoreceptor ciliary protein (Beltran et al., 2012). Subretinal injection of AAV-*RPGR* in the canine models resulted in structural and functional photoreceptor preservation. AAV-*RPGR* therapy in XLRP patients is now in phase 3 clinical trials (NCT04671433, NCT04794101). Lhériteau et al. (2014) evaluated AAV gene therapy in the *RPGRIP1*-deficient dog model of CRD/LCA. Subretinal injection of AAV-*RPGRIP1* improved photoreceptor survival and functional rescue. AAV-*RPGRIP1* therapies have since been optimized further now with a late-stage preclinical product that utilizes an ancestral AAV serotype with broad tissue tropism (Wassmer et al., 2017). More recently, Aguirre et al. showed that AAV-*NPHP5* therapy in *NPHP5* mutant dogs stably restores photoreceptor structure, function, and vision (Aguirre et al., 2021). The safety and efficacy of gene therapy in these large animal ciliopathy models of IRDs provide a path for translation to human treatment.

### 5.3 Optimal therapeutic target in the ciliary complex

AAV-based gene delivery approach has been studied extensively in the emerging molecular therapies against photoreceptor ciliopathies. Clinical trials are ongoing, including those aimed at augmenting the genes such as *RPGR*, *RPGRIP1*, *NPHP4*, or *NPHP5*. Notably, these examples are monogenic IRDs where a single gene defect is causal hence its augmentation was expected to be curative. Increasingly, IRDs arising from defects in more than one gene are being recognized and molecularly characterized among patients as well as in animal models (Appelbaum et al., 2020). In a canine model of *RPGRIP1*-CRD, multiple loci, two of which correspond to genes encoding ciliary proteins RPGRIP1 and MAP9, have been associated with disease expression (Das et al., 2017b), potentially obscuring the optimal therapeutic target. Detailed phenotypic evaluation of mutant canines variably affected by the loci confirmed the primary disease loci as *RPGRIP1* (Ripolles-Garcia et al., 2023) indicating that it would be the solely sufficient therapeutic target, rather than necessitating multiple genes to be augmented. The complexes formed by ciliary proteins at each functional site of the ciliary structure make the corresponding disease phenotypes susceptible to modifications by changes in their interacting proteins. Phenotypic and future preclinical studies in animal models provide insights into identifying and refining the optimal therapeutic target molecules.

## 6 Conclusion

**FIGURE 1:**
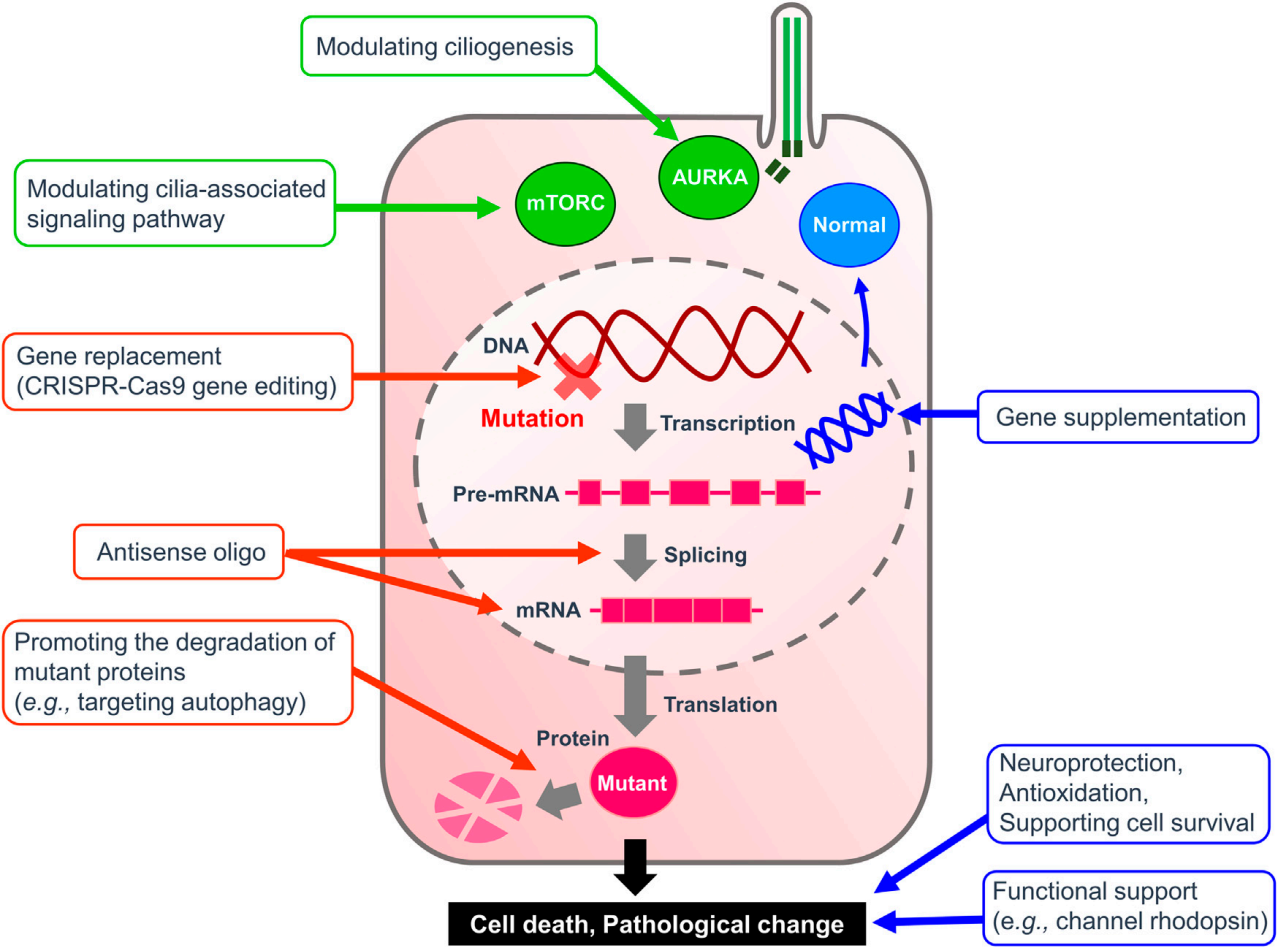
Therapeutic strategy for ciliopathies. A diagram illustrating the therapeutic targets to alleviate ciliopathies. Green arrows highlight targeting ciliogenesis and cilia-related pathways summarized in Table 2. Red arrows indicate therapies to remedy the causal mutation itself or its products. Blue arrows show the treatments to replace the malfunctioning system or to assist the survival of affected cells.

The significance of primary cilia in normal physiology and disease pathology is increasingly being recognized as our understanding of the unique characteristics of primary cilia continues to expand. Of particular interest is the localization and physiologic role of ciliary receptors that are critical in primary cilia function as sensory organelles. In addition to the US FDA-approved SMO antagonists, the development of drugs that target other receptors is desired as diagnostic, preventive, and therapeutic agents in the future. ​​Ongoing clarification of the molecular mechanistic basis of ciliary function including ciliogenesis, ciliary trafficking, and ciliary maintenance is leading to possible targets and windows for therapeutic intervention to be developed. For example, functional characterization of PJA2, a ubiquitin ligase, revealed that ubiquitylation of BBS1 by PJA2 regulates ciliary trafficking of GPR161 in hTERT-RPE1 cells (Chiuso et al., 2023). Cilia-specific ubiquitinome analysis identified proteins that could regulate ESCRT-dependent clathrin-mediated endocytosis and caveolin 1-mediated cilia formation in murine inner medullary collecting duct 3 (IMCD3) cells and hTERT-RPE1 cells, respectively (Aslanyan et al., 2023). *In silico* approach using the International Mouse Phenotype Consortium data and STRING, a database of known and predicted protein-protein interactions, have successfully found novel ciliopathy genes (Higgins et al., 2022). Chemicals to regulate these ciliary genes could be generated using technologies that lead to targeted protein degradation, such as proteolysis-targeting chimeras and small-molecule hydrophobic tagging (Bhole et al., 2023; Xie et al., 2023). *In vitro* phenotypic screening can also be used for repositioning clinical drugs to treat ciliopathies (Benmerah et al., 2023). For example, eupatilin, a drug used to treat gastritis and peptic ulcers, was identified as a positive hit that could induce ciliogenesis in a drug screening using CEP290-null cells (Kim et al., 2018). Recent studies have also revealed that primary cilia are involved in a wide variety of cellular functions such as the regulation of spliceosome and the facilitation of signaling (Cerulo et al., 2023; Macarelli et al., 2023), suggesting that chemicals targeting primary cilia may be applicable to treat an array of diseases than previously thought. To date, multiple examples of ciliopathy IRD gene therapy in large animal models of retinal degeneration have shown that ciliopathy phenotypes may be reversed. At present, the therapeutic strategy for the retinal ciliiopathies remain largely aimed at rescuing or restoring the function of the target cell as a whole, rather than focusing on a particular mechanistic process or subceullar structure associated specifically with the cilia (Figure 1). Owing to the considerable advances in the field of viral gene delivery and model systems, development of gene therapies that augment molecules critical in the function and structure of photoreceptor cilia such as CEP290 and RPGR have exploded in recent years (Table 4). Further advances in both *in vitro* and *in vivo* studies are expected to bring targeted therapy to patients suffering from various forms of ciliopathies.

**TABLE 4:** Clinical trials in ciliopathies featuring retinal dystrophies.

Retinal dystrophies	Associated gene mutations	Intervention/Description		CT.gov_identifier
Leber Congenital Amaurosis (LCA)	*CEP290,* c.2991 + 1655A>G Mutation (p.Cys998X)	Sepofarsen (QR-110)	A splice-modulating oligonucleotide	NCT04855045
		EDIT-101	A gene editing drug by CRISPR-Cas9	NCT03872479
Retinitis Pigmentosa (RP)	RHO	ZVS203e	rAAV-mediated gene editing drug that silences RHO mutant protein expression by CRISPR/Cas9	NCT05805007
	*RHO* (P23H)	QR-1123	An antisense oligonucleotide, designed to specifically target the mutant P23H mRNA	NCT04123626
		BS01	A recombinant AAV-based gene therapy expressing an enhanced light-sensitive channelrhodopsin gene that is targeted to the optic nerve	NCT04278131
		RST-001	An intravitreal AAV2 vector to transfer Channelrhodopsin-2 (ChR2) to retinal ganglion cells	NCT02556736
		KIO-301	A light-sensing small molecule designed to reactivate visual function of the eye in response to light	NCT05282953
	*RHO* (P23H)	ADX-2191	An intravitreal formulation of methotrexate, which promotes P23H rhodopsin degradation	NCT05392179
	*RHO* (P23H)	Hydroxychloroquine (HCQ)	HCQ may arrest progression of retinal degeneration by altering the autophagy pathway in photoreceptors	NCT04120883
		*N*-acetylcysteine (NAC)	NAC reduces oxidative stress and in animal models of RP it slowed cone degeneration	NCT05537220
		EA-2353	A first-in-class small molecule that selectively activates endogenous retinal stem and progenitor cells	NCT05392751
LCA or RP		Human primary Retinal Pigment Epithelial (HuRPE) Cells Subretinal Transplantation		NCT03566147
RP or other retinal dystrophies		An autologous bone marrow-derived stem/progenitor cells administered intravitreously		NCT03772938
RP Associated With Usher Syndrome	*USH2A*	QR 421a (ultevursen)	An RNA therapy promoting *USH2A* exon 13 skipping	NCT05085964
		NPI-001	A GMP-grade of *N*-acetylcysteine amide (NACA)	NCT04355689
X-Linked RP	*RPGR*	AAV5-RPGR	An AAV-based gene therapy expressing RPGR	NCT04794101, NCT04671433
	*RPGR*	4D-125 IVT	A gene replacement therapy for XLRP	NCT04517149
	*RPGR*	BIIB112	An AAV-based gene therapy expressing RPGR	NCT03116113

## Figures and Tables

**Table:** 

**AAV**	Adeno-associated virus
**AC**	Adenylyl cyclase
**AurA**	Aurora A kinase
**Arl13B**	ADP-ribosylation factor-like 13B
**BBS**	Bardet-Biedl syndrome
**CD**	Cone degeneration
**CEP55**	Centrosomal protein 55
**CP110**	Coiled-coil protein 110
**CRD**	Cone-rod dystrophy
**CTSs**	Ciliary targeting sequences
**DUB**	Deubiquitinases
**ER**	Endoplasmic reticulum
**ESCRT**	Endosomal sorting complex required for transport
**FFAR**	Free-fatty acid receptor
**GPCR**	G protein-coupled receptor
**GSK3β**	Glycogen synthase kinase 3β
**HDAC6**	Histone deacetylase 6
**5-HT6**	Serotonin receptor 6
**IFT**	Intraflagellar transport
**IGF-1R**	Insulin-like growth factor-1 receptor
**IMCD cells**	Inner medullary collecting duct cells
**INPP5E**	Inositol polyphosphate-5-phosphatase
**IQCB1**	IQ motif containing B1
**IRD**	Inherited retinal disease
**KISS1R**	Kisspeptin receptor 1
**LCA**	Leber congenital amaurosis
**MAP9**	Microtubule associated protein 9
**MARCHF7**	Membrane associated ring-CH-type finger 7
**MC4R**	Melanocortin 4 receptor
**mTOR**	Mammalian target of rapamycin
**mTORC**	MTOR complex
**MCHR1**	Melanin-concentrating hormone receptor 1
**NEDD9**	Neural precursor cells expressed, developmentally downregulated protein 9
**Nek2**	NIMA-related protein kinase 2
**NPHP4**	Nephrocystin 4
**NPHP5**	Nephrocystin 5
**NPY**	Neuropeptide Y receptors
**OS**	Outer segment
**PDGFRα**	Platelet-derived growth factor receptor α
**PDK1**	Phosphatidylinositol-dependent protein 1
**PKA**	Protein kinase A
**PIP2**	Phosphatidylinositol 4,5-bisphosphate
**PIP3**	Phosphatidylinositol 3,4,5-trisphosphate
**PLK1**	Polo-like kinase 1
**PTHR**	Parathyroid hormone receptor
**PTCH1**	Patched-1
**RP**	Retinitis pigmentosa
**RPGR**	Retinitis pigmentosa GTPase regulator
**RPGRIP1**	Retinitis pigmentosa GTPase regulator interacting protein 1
**RTK**	Receptor tyrosine kinase
**SEPT2**	Septin 2
**SMO**	Smoothened
**SSTR3**	Somatostatin receptor 3
**STK36**	Serine/threonine protein kinase 36
**TCHP**	Trichoplein
**TEM**	Transmission electron microscopy
**TGF-βR**	Transforming growth factor-β receptor
**TGN**	Trans-Golgi network
**TRIM32**	Tripartite motif containing 32
**TSC1/2**	Tuberous sclerosis complex 1/2
**TULP3**	Tubby-like protein 3
**ULK3**	Unc-51 like kinase 3
**UPS**	Ubiquitin-proteasome system
**USP8**	Ubiquitin specific peptidase 8
**USP9X**	Ubiquitin specific peptidase 9X
**USP33**	Ubiquitin specific peptidase 33
**XLRP**	X-linked retinitis pigmentosa
